# Machine Learning in Left Ventricular Hypertrophy Detection: Systematic Review and Meta-Analysis

**DOI:** 10.2196/76637

**Published:** 2026-02-27

**Authors:** Yilin Li, Ke Zhao, Jing Wu

**Affiliations:** 1 Department of Geriatrics, The Third People’s Hospital of Chengdu, 82 Qinglong Street, Qingyang District, Chengdu, Sichuan Province, China, 610031, Chengdu, Sichuan, China, 86 15881707332; 2 Department of Critical Care Medicine, Affiliated Hospital of North Sichuan Medical College, Nanchong, Sichuan, China

**Keywords:** artificial intelligence, AI, machine learning, left ventricular hypertrophy, meta-analysis, deep learning, electrocardiogram, ECG, echocardiography, cardiovascular risk

## Abstract

**Background:**

In recent years, researchers have investigated machine learning (ML)–based approaches for the detection of left ventricular hypertrophy (LVH). However, the accuracy of ML in detecting LVH varies across different modeling variables and models. Systematic evidence is lacking in understanding how different ML approaches affect LVH detection accuracy.

**Objective:**

The aim of this study is to systematically assess the diagnostic accuracy of these ML approaches to inform the development of artificial intelligence tools.

**Methods:**

PubMed, Embase, Cochrane Library, and Web of Science were comprehensively searched up to November 12, 2025. The Prediction Model Risk of Bias Assessment Tool was used to evaluate the risk of bias. Subgroup analyses were performed based on ML model types and modeling variables (electrocardiogram [ECG], clinical features, and echocardiography). Only diagnostic 2×2 tables from validation sets were pooled for meta-analysis, with all statistical analyses performed using Stata.

**Results:**

A total of 25 studies were included in the analysis. The performance of ML models varied with input data types and algorithms. A meta-analysis showed that ECG-based models, in comparison, exhibited a sensitivity of 0.76 (95% CI 0.66‐0.84) and a specificity of 0.84 (95% CI 0.78‐0.89). Echocardiography-based models had a sensitivity ranging from 0.71 to 0.94 and a specificity ranging from 0.67 to 0.96. The models based on clinical features had a sensitivity of 0.78 (95% CI 0.69‐0.85) and a specificity of 0.71 (95% CI 0.65‐0.76). A subgroup analysis of the ECG-based models revealed that the deep learning model produced a sensitivity of 0.71 (95% CI 0.60‐0.80) and a specificity of 0.79 (95% CI 0.65‐0.88).

**Conclusions:**

ML demonstrates reasonably high accuracy in detecting LVH. However, these conclusions are derived from limited evidence. Meanwhile, the extreme heterogeneity reported in the meta-analysis requires more critical interpretation. Current conclusions regarding model accuracy should be interpreted with caution. Therefore, future research should focus on constructing high-performance ML models based on imaging data for LVH diagnosis.

## Introduction

Left ventricular hypertrophy (LVH) represents an increase in left ventricular mass driven by various cardiovascular risk factors [1], and it confers a 5‐10 times greater risk of developing cardiovascular disease [2,3]. Accumulating evidence demonstrates that LVH is an independent predictor of cardiovascular risk [4-6]. However, the pathological and physiological alterations induced by LVH occur without overt symptoms or clinical signs [1]. Therefore, the early detection of LVH through sensitive screening methods is imperative, as it holds significant positive implications for patient health outcomes.

Currently, echocardiography (ECHO) and electrocardiogram (ECG) are the conventional diagnostic methods for detecting LVH [7,8]. While ECHO is a relatively accurate approach [9], it is costly and requires specialized equipment and trained operators, which poses challenges for early diagnosis [9,10]. Studies also show high rates of disagreement in ECHO readings among cardiologists, sometimes as much as 42.1% [11]. Cardiologists may mistakenly read ECHOs, particularly when tired, possibly resulting in misdiagnoses and misinterpretations [12,13]. Machine learning (ML) has garnered increasing attention in the medical field over the past decade, particularly in cardiovascular medicine [14-16]. ML is important since it can discern critical features from complex datasets [17].

Given its capacity to process intricate, high-volume health data, ML has ascended as an indispensable tool for developing predictive models in medicine [17,18]. In this context, researchers have begun constructing ML-based approaches for diagnosing LVH.

Nonetheless, there are various models and modeling variables for constructing ML models [17,19,20]. Multiple ML models have been proposed for detecting LVH [1,10,21,22], yet their comparative diagnostic accuracy remains debated. Robust evidence assessing both the accuracy and real-world implementation of ML models for LVH identification remains insufficient. Therefore, a systematic review and meta-analysis was conducted in this study to evaluate the performance of ML in detecting LVH and furnish empirical support for artificial intelligence (AI) development in this field.

## Methods

### Study Registration

This study followed the PRISMA (Preferred Reporting Items for Systematic Reviews and Meta-Analyses) guidelines and was prospectively registered in PROSPERO (registration ID: CRD42024617183) [23].

### Eligibility Criteria

Detailed inclusion and exclusion criteria were formulated for this review. To improve the visualization effect, these standards were presented in tabular form (Textbox 1).

Textbox 1. **Inclusion and exclusion criteria.**

**Inclusion criteria**

- Population: individuals suspected of having left ventricular hypertrophy
- Intervention: machine learning (ML) models for left ventricular hypertrophy identification
- Comparison: no diagnostic tool was used as a comparator
- Outcome: quantitative diagnostic performance metrics, including C-statistic, sensitivity, specificity, accuracy, recall, precision, confusion matrices, diagnostic 2×2 tables, *F*_1_-score, or calibration curves
- Study design: case-control, cohort, or cross-sectional studies published in English

**Exclusion criteria**

- Intervention: studies that only examined differential factors without developing complete ML models
- Outcomes: studies lacking the following metrics for ML performance: C-statistic, sensitivity, specificity, accuracy, recall, precision, confusion matrices, diagnostic 2×2 tables, *F*_1_-score, or calibration curves
- Study design: meta-analyses, reviews, guidelines, expert opinions, and unpublished conference abstracts

### Data Sources and Search Strategy

PubMed, Embase, Cochrane Library, and Web of Science were systematically searched up to November 12, 2025, using both Medical Subject Headings (MeSH) terms and free-text words. Search terms included combinations of “artificial intelligence,” “deep learning,” “machine learning,” “random forest,” “support vector machine,” and “left ventricular hypertrophy.” No filters were applied to constrain studies by publication year or geographic region. The details on the search strategy are available in Multimedia Appendix 1.

### Study Selection and Data Extraction

All identified records were uploaded to EndNote, and duplicates were filtered out. The titles and abstracts of the remaining studies were screened for potentially relevant studies. Full texts of the selected studies were obtained and evaluated for eligibility. An electronic extraction sheet was prepared in advance to record author information, publication year, country of the author, study design, patient sources, generation method of validation set, model types, and modeling variables. The entire screening process and data extraction were performed independently by 2 investigators, followed by cross-verification. Discrepancies were addressed through discussion with a third researcher.

### Risk of Bias in Studies

The Prediction Model Risk of Bias Assessment Tool (PROBAST) was leveraged to appraise the risk of bias (RoB) in the included original studies [24]. The evaluation covers 4 domains: participants, predictor variables, outcomes, and statistical analysis, which collectively determine the overall RoB and applicability. Each domain contains specific signaling questions (2, 3, 6, and 9 questions, respectively), with 3 possible responses: “yes/probably yes” (low RoB), “no or probably no” (high RoB), and “no information” (unclear). Two researchers independently conducted the PROBAST assessments, followed by cross-checking. Any dissents were addressed through discussion with a third researcher.

### Synthesis Methods

Some studies had multiple validation sets. When both an internal and an external validation set were available, only the results from the external set were retained. If an external validation set is unavailable, the results from the internal validation set with optimal performance were selected.

A meta-analysis was conducted on the validation set, leveraging diagnostic 2×2 tables. For studies without 2×2 tables, sensitivity, specificity, positive predictive value, and accuracy, combined with case numbers, were used for estimation (formulas 1‐5). The bivariate mixed-effects model was used to pool diagnostic metrics (sensitivity, specificity, positive likelihood ratio [PLR], negative likelihood ratio [NLR], diagnostic odds ratio [DOR], and summary receiver operating characteristic [SROC]). Deek funnel plot was used for assessing publication bias [25], and Fagan nomogram was adopted for evaluating clinical utility [26]. Subgroup analyses were carried out based on modeling variables and model types. A significance threshold of *P*<.05 was established.

TP=Sensitivity×Events (1)

TN=Specificity×(samplesize − Events) (2)

FN=Events-TP (3)

FP=samplesize-Events-TN (4)

OR

FP=TP/precision-TP (5)

## Results

### Study Selection

**Figure 1.**
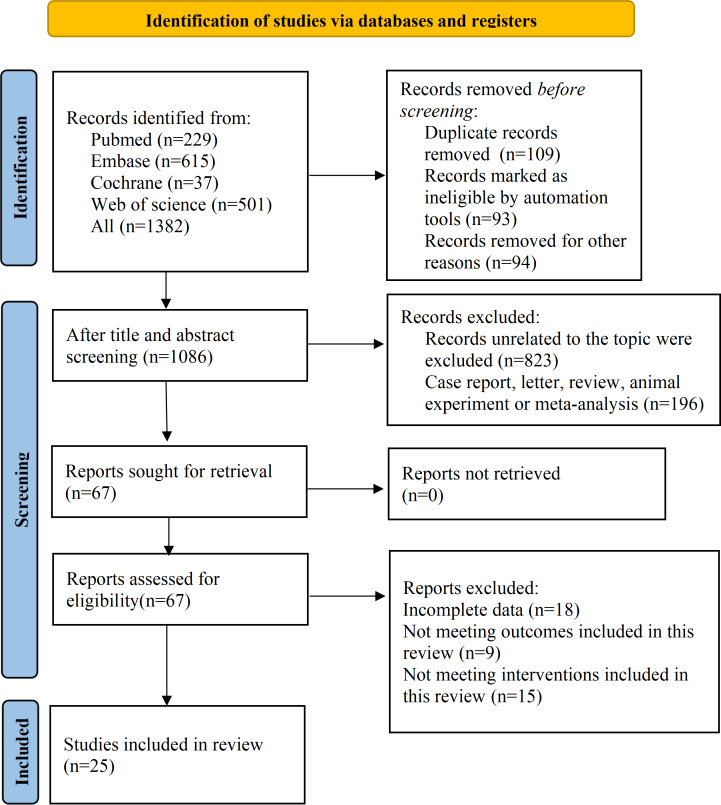
PRISMA (Preferred Reporting Items for Systematic Reviews and Meta-Analyses) flowchart for literature screening.

A total of 1382 records were searched. After removing duplicates, 1086 records remained. Then, 1019 records were excluded through title and abstract screening, leaving 67 records for full-text review. Of those, 42 reports were removed due to incomplete data, incompatible outcomes, and incompatible interventions. Ultimately, 25 studies were selected for analysis (Figure 1).

### Study Characteristics

Of the 25 case-control studies included (2018‐2025) [1,10,21,22,27-47], 19 used single-center designs, and 6 adopted multicenter approaches (including 4 studies based on databases that leveraged the UK Biobank resource). These studies were conducted across diverse regions: 10 studies in China, 6 in the United States, 2 in Japan, 2 in Korea, 1 in Mexico, 1 in the United Kingdom, 1 in Greece, 1 in Italy, and 1 in the United Arab Emirates. ECG-based models were included in 16 studies, clinical feature-based models were included in 5 studies, and ECHO-based models were included in 4 studies. Six studies performed external validation, including 5 ECG-based models and 1 clinical feature-based model. The main features of these studies are summarized in Table 1.

**Table 1.** Basic features of the included studies.

Author and publication year	Country of author	Study design	Patient source	Validation set generation method	Model type	Modeling variable	Reference standard
Ryu et al [22] (2023)	Korea	Case-control study	Single center	RS^a^	DL^b^	12-lead ECG^c^	ECHO^d^
Farhad et al [28] (2023)	United Arab Emirates	Case-control study	LVH^e^ database	RS 10 folds	DL	ECHO	Clinical consensus
Zhao et al [32] (2022)	China	Case-control study	Single center	Folds RS	DL	12-lead ECG	Clinical consensus
Liu et al [33] (2022)	China	Case-control study	Multicenter	Folds EV^f^	DL	12-lead ECG	Clinical consensus
De la Garza Salazar et al [45] (2021)	Mexico	Case-control study	Single center	RS EV	DT^g^	12-lead ECG	Clinical consensus
Kwon et al [40] (2020)	Korea	Case-control study	Multicenter	RS EV	DL	12-lead ECG	Clinical consensus
Ghorbani et al [39] (2020)	United States	Case-control study	Single center	RS	DL	ECHO	Clinical consensus
Sparapani et al [43] (2019)	United States	Case-control study	The MESA^h^ database	RS	DT	12-lead ECG	CMR^i^
Madani et al [44] (2018)	United States	Case-control study	Single center	RS	DL	ECHO	Clinical consensus
Yuan et al [29] (2023)	China	Case-control study	Single center	RS	LR^j^	Clinical features	Clinical consensus
Dwivedi et al [30] (2023)	United States	Case-control study	Single center	RS	RF^k^	12-lead ECG	ECHO
Yu et al [35] (2022)	China	Case-control study	Single center	RS	DL	ECHO	Clinical consensus
Wu et al [34] (2022)	China	Case-control study	Single center	EV	LR	Clinical features	Clinical consensus
Kokubo et al [36] (2022)	Japan	Case-control study	Single center	RS	DL	12-lead ECG	Clinical consensus
Ye et al [37] (2021)	China	Case-control study	Single center	10 folds^l^	LASSO^m^ regression	Clinical features	Clinical consensus
Angelaki et al [38] (2021)	Greece	Case-control study	Single center	RS	RF	12-lead ECG	Clinical consensus
Lin and Liu [41] (2020)	United States	Case-control study	Single center	RS 4 folds	SVM^o^	12-lead ECG	ECHO
Tison et al [42] (2019)	United States	Case-control study	Single center	RS	GBM^n^	12-lead ECG	ECHO
Liu et al [31] (2023)	China	Case-control study	Single center	RS	ANN^p^	12-lead ECG	Clinical consensus
Zhang et al [1] (2024)	China	Case-control study	Single center	RS	LR	Clinical features	Clinical consensus
Wan et al [27] (2024)	China	Case-control study	Single center	RS	LR	Clinical features	Clinical consensus
Naderi et al [21] (2024)	United Kingdom	Case-control study	UK Biobank Database+ Single center	RS EV	SVM	12-lead ECG	CMR
Cai et al [10] (2024)	Japan	Case-control study	Single center	RS	DL	12-lead ECG	Clinical consensus
Huang et al [46] (2025)	China	Case-control study	Single center	RS	CatBoost	12-lead ECG	Clinical consensus
Taconné et al [47] (2025)	Italy	Case-control study	PTB-XL ECG database+ Georgia 12-lead ECG Challenge Database	10 folds EV	SVM	12-lead ECG	Clinical consensus

a RS: random sampling.

b DL: deep learning.

c ECG: electrocardiography.

d ECHO: echocardiography.

e LVH: left ventricular hypertrophy.

f EV: external validation.

g DT: decision tree.

h MESA: Multi-Ethnic Study of Atherosclerosis.

i CMR: cardiac magnetic resonance.

j LR: logistic regression.

k RF: random forest.

l 10 folds: 10-fold cross-validation.

m LASSO: least absolute shrinkage and selection operator.

n GBM: gradient boosted machine.

o SVM: support vector machine.

p ANN: artificial neural network.

### RoB in Studies

**Figure 2.**
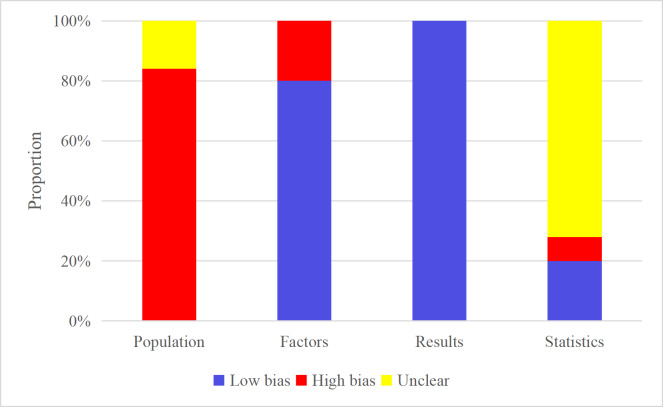
Risk of bias of the included machine learning models.

A high RoB was identified in 21 case-control studies (single- and multi-center) due to potential selection bias. An unclear RoB was assigned to 4 studies based on public databases. The 21 case-control studies were rated as having a high RoB due to selection bias. Due to inherent selection biases, such as the “healthy volunteer” effect commonly found in biobanks, the 4 studies based on public databases were rated as having an unclear RoB. Five case-control studies utilized clinical features for modeling and were rated as having a high RoB due to case-control influence during data collection. The remaining studies, which used image-based ML models, were assessed as having a low RoB in terms of predictors. All studies used valid outcome definitions, and the avoidance of predictor variables in model construction ensured a low RoB. For the imaging-based validation set, the accurate calculation of events per variable (EPV) proved challenging, leading to an uncertain RoB. Developers typically rely on EPV, especially EPV of 10 higher, to determine the minimum sample size required and the maximum number of candidate predictions that can be tested [48]. However, when building models based on images, the basic unit of validation data is the image, not the traditional “patient case.” Therefore, we cannot calculate the sample size that meets the EPV requirements based on the number of patient cases, making it difficult to directly apply this standard in such studies. In contrast, studies using clinical features for modeling consistently met the EPV>10 criterion and thus were classified as low RoB. In 2 studies, data were segmented according to images, and patients’ images were shared between the training set and the validation set, which may lead to data leakage, and thus were classified as high RoB (Figure 2).

### Meta-Analysis

#### ECG-Based ML Model for LVH Detection

**Figure 3.**
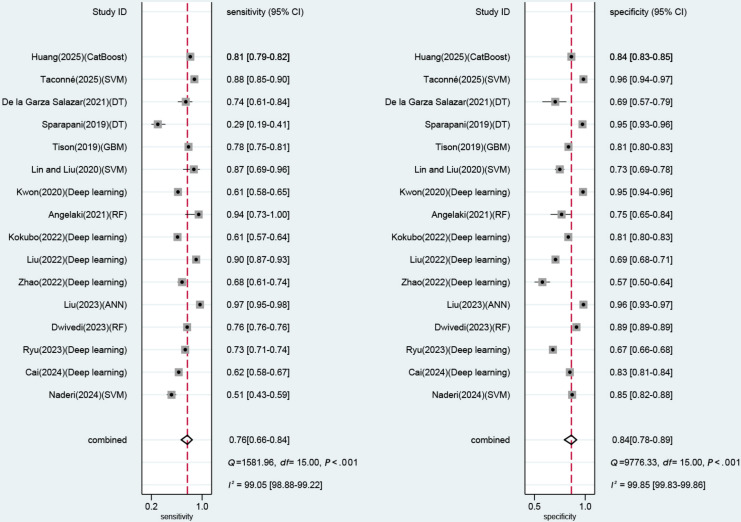
Forest plot of sensitivity and specificity for electrocardiogram-based models [10,21,22,30-33,36,38,40-43,45-47]. ANN: artificial neural network; DT: decision tree; GBM: gradient boosting machine; RF: random forest; SVM: support vector machine.

In the validation set, the performance of ECG-based ML models was evaluated in 16 diagnostic 2×2 tables. The meta-analysis results demonstrated the following estimates: sensitivity 0.76 (95% CI 0.66‐0.84), specificity 0.84 (95% CI 0.78‐0.89), PLR 4.8 (95% CI 3.3‐7.0), NLR 0.28 (95% CI 0.19‐0.41), DOR 17 (95% CI 9‐33), and SROC 0.88 (95% CI 0.75‐0.95; Figure 3 and Figure S1 in Multimedia Appendix 2). The Deek funnel plot showed no significant publication bias among the included studies (*P*=.43; Figure S2 in Multimedia Appendix 2). Given a pretest high-risk probability of 10%, if the model result indicated LVH, the posterior probability of LVH was 35%; if the model result indicated non-LVH, the posterior probability of LVH was 3% (Figure S3 in Multimedia Appendix 2).

Subgroup analyses were performed across various ML approaches. The deep learning (DL) model produced a sensitivity of 0.71 (95% CI 0.60‐0.80) and a specificity of 0.79 (95% CI 0.65‐0.88).

Subgroup analyses were performed across various validation approaches. The results showed that internal validation produced a sensitivity of 0.77 (95% CI 0.64‐0.86) and a specificity of 0.83 (95% CI 0.75‐0.89), while external validation produced a sensitivity of 0.76 (95% CI 0.60‐0.87) and a specificity of 0.87 (95% CI 0.74‐0.94; Table 2).

**Table 2.** Subgroup analyses of electrocardiography (ECG)–based machine learning (ML) models across various validation approaches.

Methods	Machine learning approaches, n	Sen^a^ (95% CI)	Spe^b^ (95% CI)	PLR^c^ (95% CI)	NLR^d^ (95% CI)	DOR^e^ (95% CI)	SROC^f^ (95% CI)
IV^g^	11	0.77 (0.64‐0.86)	0.83 (0.75‐0.89)	4.5 (2.9‐6.8)	0.28 (0.17‐0.46)	16 (7-35)	0.87 (0.74‐0.94)
EV^h^	5	0.76 (0.60‐0.87)	0.87 (0.74‐0.94)	5.8 (2.8‐12.2)	0.28 (0.16‐0.48)	21 (7-60)	0.89 (0.52‐0.98)
Overall	16	0.76 (0.66‐0.84)	0.84 (0.78‐0.89)	4.8 (3.3‐7.0)	0.28 (0.19‐0.41)	17 (9-33)	0.88 (0.75‐0.95)

a Sen: sensitivity.

b Spe: specificity.

c PLR: positive likelihood ratio.

d NLR: negative likelihood ratio.

e DOR: diagnostic odds ratio.

f SROC: summary receiver operating characteristic.

g IV: internal validation.

h EV: external validation.

As for internal validation, the meta-analysis results demonstrated an SROC of 0.87 (95% CI 0.74‐0.94; Figure S4 in Multimedia Appendix 2). The Deek funnel plot showed no significant publication bias among the included studies (*P*=.30; Figure S5 in Multimedia Appendix 2). Given a pretest high-risk probability of 10%, if the model result indicated LVH, the posterior probability of LVH was 33%; if the model result indicated non-LVH, the posterior probability of LVH was 3% (Figure S6 in Multimedia Appendix 2).

As for external validation, the meta-analysis results showed an SROC of 0.89 (95% CI 0.52‐0.98; Figure S7 in Multimedia Appendix 2). The Deek funnel plot showed no significant publication bias across the included studies (*P*=.37; Figure S8 in Multimedia Appendix 2). Given a pretest high-risk probability of 10%, if the model result indicated LVH, the posterior probability of LVH was 39%; if the model result indicated non-LVH, the posterior probability of LVH was 3% (Figure S9 in Multimedia Appendix 2).

Dwivedi et al [30] used image-level splitting, which involved sharing patient images between training and validation. This method posed a high RoB due to data leakage. Accordingly, a sensitivity analysis was conducted on the ECG-based ML model group to exclude this study. The meta-analysis results demonstrated the following estimates: sensitivity 0.76 (95% CI 0.66‐0.85), specificity 0.84 (95% CI 0.77‐0.89), PLR 4.7 (95% CI 3.2‐7.0), NLR 0.28 (95% CI 0.19‐0.42), DOR 17 (95% CI 8‐33), and SROC 0.88 (95% CI 0.74‐0.94).

#### Clinical Feature–Based ML Model for LVH Detection

**Figure 4.**
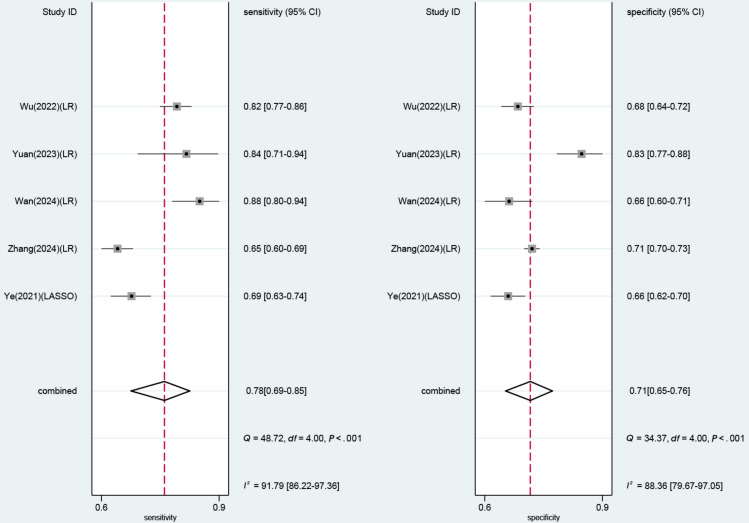
Forest plot of sensitivity and specificity for clinical feature-based models [1,27,29,34,37]. LASSO: least absolute shrinkage and selection operator; LR: logistic regression.

In the validation set, there were 5 diagnostic 2×2 tables for evaluating the performance of ML models based on clinical features. The meta-analysis results demonstrated the following estimates: sensitivity 0.78 (95% CI 0.69‐0.85), specificity 0.71 (95% CI 0.65‐0.76), PLR 2.7 (95% CI 2.1‐3.4), NLR 0.31 (95% CI 0.21‐0.46), DOR 9 (95% CI 5‐16), and SROC 0.79 (95% CI 0.62‐0.90; Figure 4 and Figure S10 in Multimedia Appendix 2). The Deek funnel plot showed no significant publication bias among the included studies (*P*=.09; Figure S11 in Multimedia Appendix 2). Given a pretest high-risk probability of 10%, if the model result indicated LVH, the posterior probability of LVH was 23%; if the model result indicated non-LVH, the posterior probability of LVH was 3% (Figure S12 in Multimedia Appendix 2).

#### ECHO-Based ML Model for LVH Detection

Farhad et al [28] used image-level splitting, which involved sharing patient images between training and validation. This method posed a high RoB due to data leakage. Accordingly, a sensitivity analysis was conducted in the ECHO-based ML model group to exclude this study. Ghorbani et al [39] developed a DL model based on ECHO. In their validation set of 373 cases (including 142 LVH cases), the model had a pooled sensitivity of 0.71 and a specificity of 0.67. Madani et al [44] also developed a DL model using echocardiographic data. Their validation set comprised 200 cases (100 LVH cases), with the model demonstrating a pooled sensitivity of 0.78 and a specificity of 0.96. Similarly, Yu et al [35] implemented a DL approach for ECHO. Their validation set included 150 cases (97 LVH cases), and the model yielded a pooled sensitivity of 0.94 and a specificity of 0.92.

## Discussion

### Principal Findings

This study examined the efficacy of 3 types of ML models for LVH detection. In the validation cohort, ECG-based models had a sensitivity of 0.76 (95% CI 0.66‐0.84) and a specificity of 0.84 (95% CI 0.78‐0.89). The clinical feature-based models had a sensitivity of 0.78 (95% CI 0.69‐0.85) and a specificity of 0.71 (95% CI 0.65‐0.76). The ECHO-based models had a sensitivity ranging from 0.71 to 0.94 and a specificity ranging from 0.67 to 0.96. These findings confirm the significant efficacy of ML in LVH detection.

### Comparison to Prior Work

ML is increasingly accurate in LVH detection, which enables the identification and treatment of earlier subclinical cardiovascular diseases [49]. In clinical practice, ECG is now widely used as a screening method for LVH due to its affordability and high accessibility, and is recommended as part of standard clinical evaluation for patients with hypertension in the Chinese Hypertension Clinical Practice Guidelines [50]. ECG-based LVH detection has been a major research priority in hypertensive cardiac target organ damage studies [1]. The clinical diagnosis of LVH via 12-lead ECG relies principally on the quantitative assessment of QRS complex (Q-wave, R-wave, S-wave) voltage parameters [51,52]. Despite recent innovations in diagnostic criteria, the overall sensitivity remains suboptimal, particularly in Chinese and other East Asian populations [1,53,54]. The low sensitivity of ECG in LVH detection limits its clinical application [32]. Consistent with existing research, our results showed that ECG-based models produced a sensitivity of 0.76 (95% CI 0.66‐0.84) and a specificity of 0.84 (95% CI 0.78‐0.89). Although limited by its sensitivity, studies have confirmed that ECG-detected LVH is associated with adverse cardiovascular outcomes [32,55]. Given its widespread availability and low cost, ECG remains an ideal initial assessment tool for LVH screening in patients with hypertension [56]. Therefore, some researchers recommend using ECG primarily for the risk stratification and prognosis prediction of LVH [43,57].

It has also been noted that different ML methods show significant differences in their ability to identify positive or outcome events. With its powerful automatic feature extraction capabilities, DL models can directly identify complex and subtle patterns from imaging data, integrate multiple imaging variables, and improve diagnostic accuracy [58,59]. Its automated segmentation reduces the subjective bias of traditional manual segmentation in radiomics and can integrate segmentation with diagnosis, offering significant advantages [60,61]. Therefore, it is imperative to prioritize the development of automated methods based on DL. Decision tree models are highly interpretable models that generate rules by recursively splitting variables in a dataset [62]. Decision tree models add a penalty term proportional to the absolute value of the feature coefficient for feature selection and model building [63]. Multiple studies have confirmed that RF models typically exhibit higher predictive performance than other ML methods because they can model nonlinear relationships and are insensitive to overfitting [64]. RF models demonstrate significant advantages in handling imbalanced datasets [65]. RF models excel at handling nonlinear and missing data, are conducive to high-dimensional data analysis, and demonstrate strong predictive performance [66]. Support vector machine (SVM) models have high predictive accuracy [67]. For structured and well-defined data, SVM models can skillfully capture the association between biomarkers and diseases through mathematical and statistical principles, thereby achieving high diagnostic accuracy [68,69]. SVM models are known for their ability to find the optimal decision boundary in high-dimensional feature spaces, resulting in effective classification based on extracted features [70,71].

ML models that incorporated clinical features demonstrated a sensitivity of 0.78 (95% CI 0.69‐0.85) and a specificity of 0.71 (95% CI 0.65‐0.76) in the validation set. Unlike traditional ECG criteria, the clinical features used to construct these models encompassed demographic characteristics (eg, sex, age) and clinical factors (eg, systolic blood pressure, duration of hypertension, and abdominal obesity). The possible reasons for using these factors are as follows.

Sex constitutes a significant determinant of LVH [1,37]. Substantial evidence has established the female sex as an independent predictor. In previous predictive models, women scored 70, while men scored 0, possibly due to the older study population, where most women had reached menopause [1]. Women exhibit higher salt sensitivity than men due to female hormonal influences, which amplifies adverse renin-angiotensin-aldosterone system effects [72,73]. Regardless of race or menopausal status, women with salt-sensitive hypertension face a higher LVH risk because this sex-specific mechanism promotes aldosterone-driven endothelial dysfunction [72,73]. This association is further exacerbated by diminished cardiovascular protection due to decreased estrogen levels in postmenopausal women [37].

Age plays a pivotal role in forecasting LVH in young patients with salt-sensitive hypertension. The evidence reveals a dual pattern: (1) patients 40 years or older demonstrate a significantly higher risk than younger populations, consistent with age-related left ventricular mass increase [1,37,74], while (2) patients with early-onset hypertension exhibit more severe cardiac damage and elevated cardiovascular mortality [74,75]. Such observations suggest that the interaction between age and the timing of hypertension requires special attention.

The development of LVH is closely associated with systolic blood pressure level and duration of hypertension. Prospective studies demonstrate that each 19 mm Hg increase in systolic blood pressure elevates the LVH risk by 49% [6], while effective systolic blood pressure control markedly reduces the disease risk in old patients [76]. These findings provide crucial evidence for clinical treatment decisions. Hypertension duration, as a cumulative exposure metric, has been consistently validated in multiple studies to be significantly associated with LVH. Shortening the course of hypertension can effectively decrease the risk of myocardial remodeling, which shows the importance of early intervention in delaying LVH progression [77].

Obesity constitutes an independent risk factor for LVH [78,79]. Waist circumference, the principal diagnostic metric for abdominal obesity, is linked with both cardiovascular and metabolic disorders [80]. The pathophysiological linkage between abdominal obesity and LVH can be attributed to the robust correlation between waist circumference and excessive visceral adipose tissue accumulation [80,81]. As a metabolically active depot, visceral adipose tissue exhibits substantially higher energy demands compared to subcutaneous fat [81]. This augmented metabolic requirement exacerbates cardiac workload in patients with hypertension, ultimately resulting in LVH [78,81]. Studies have identified waist circumference as a superior predictor of cardiovascular risk and mortality compared to BMI [80,82]. Notably, elevated systolic blood pressure and prolonged hypertension duration may synergistically accelerate concentric cardiac remodeling, while abdominal obesity plus aging compounds metabolic risk.

The complex interactions among clinical features were quantified and integrated into ML models. While previous studies have confirmed the underlying mechanism of individual factors, models combining features from diverse dimensions provide more complete predictive insights. Specifically, sex-specific risk mechanisms dynamically interact with age-related cardiovascular changes, while blood pressure’s temporal cumulative effects and the spatial distribution of metabolic factors form a 3D LVH prediction network. This finding provides a theoretical foundation for developing clinical feature–based ML tools for precise prediction.

ECHO serves as the primary imaging tool for LVH identification. However, its clinical application faces multiple technical challenges, including a relatively low signal-to-noise ratio, inconsistent image quality, and suboptimal reproducibility [28,35]. These limitations are particularly pronounced in patients with obesity due to enhanced chest wall and subcutaneous tissue thickness, which significantly impede image acquisition [28,35]. ML technology offers a novel approach to overcoming these technical barriers. ML models incorporating ECHO demonstrated a sensitivity range of 0.71 to 0.94 and a specificity range of 0.67 to 0.96.

Existing hospital ECHO systems are integrated with this technology to enable automated image analysis and real-time LVH diagnosis. Without replacing existing manual verification procedures, it markedly increases diagnostic efficiency and proves particularly beneficial as a clinical decision aid for less experienced physicians [28]. In contemporary practice, LVH detection chiefly relies on ECHO, where ML proves to be powerful in image processing stages [35,83]. Conventional approaches that depend on manual segmentation and feature extraction are prone to information loss, whereas DL models incorporate image processing, texture extraction, and feature selection throughout the modeling process, which better preserves original image information [35,84,85]. All ML models based on ECHO in this study were DL models. Beyond improved diagnostic efficiency, ML models can automatically identify high-risk cases and enable cardiologists to prioritize urgent interventions, thus shortening care pathways and improving outcomes [49]. Additionally, this technique can enhance diagnostic capabilities at primary care levels, optimize the allocation of cardiovascular imaging resources, reduce unnecessary tests, and be significantly cost-effective in population screening and subclinical disease management [28].

Despite the superior image-processing capabilities of DL models, their clinical integration with ultrasound systems must overcome real-world challenges, including real-time performance, device compatibility, and patient data privacy. Furthermore, the stability of ML models across demographics needs further validation in multicenter research. Future research should prioritize enlarging and diversifying ECHO image databases and validating the actual performance of ML in the detection of LVH in prospective trials.

Meta-analysis of ECG-based ML models for detecting LVH revealed substantial heterogeneity. This restricts the statistical reliability of pooled performance metrics, preventing their interpretation as definitive generalization conclusions. Potential sources of such heterogeneity are manifold. Methodologically, substantial variation in ECG signal preprocessing, feature extraction, and used algorithms introduces fundamental differences. Clinically, diverse underlying disease profiles and severity among studied populations affect model consistency. Regional demographic variations further increase result dispersion. Consequently, pooled estimates from our research require cautious interpretation. Their value lies more in demonstrating the broad performance range and limited confirmatory nature of current evidence than in providing a stable performance expectation. Future efforts should prioritize methodological standardization and prospective multicenter validation to enhance evidence quality in this domain.

When the PROBAST was used to assess model quality, we found that the results raised some concerns, particularly in terms of its strict evaluation of the study population. We believe that some of its criteria may be overly stringent. Although the PROBAST is suitable for evaluating multivariate models for diagnosis and prediction, it is primarily based on retrospective case-control studies, with few prospective studies included [86]. This design preference often leads to a high risk of bias in assessment results, which poses a significant challenge to diagnostic model research. In the studies we included, most involved diagnostic models, and the case selection process itself might introduce bias. The PROBAST uses the EPV principle for sample size estimation, but image-based ML models face challenges in applying EPV rules for bias risk assessment. In future studies, we may explore prior sample size estimation during the research design phase, leveraging ML-based expected performance metrics, such as area under the curve and its 95% CI. Moreover, learning curve analysis serves as a powerful empirical tool. By plotting how a model’s performance on both the training and validation sets changes as the sample size increases, we can intuitively determine whether the model’s performance has reached a plateau, thereby establishing the optimal number of cases. In the field of DL, there exist several widely accepted rules. For instance, in complex image classification tasks, it is generally recommended that each category requires at least several thousand or even tens of thousands of training images to achieve good generalization performance. Although these guidelines are somewhat rough, they provide a practical benchmark for assessing sample sizes in large-scale imaging studies. In addition, the PROBAST requires the verification of the consistency between model statistical weights and actual reported results, which is particularly difficult for models with low interpretability (such as SVM), as they typically do not provide the weight information required in the original validation set [24]. These issues indicate that while the PROBAST is widely used for bias risk assessment in ML research, the observed limitations arise more fundamentally from the inherent biases of the retrospective case-control design—which is applied by most of the current ML diagnostic studies—than from an inherent excessive stringency in the PROBAST criteria. Advancing this field requires constructing and validating models through prospective, multicenter, double-blinded cohort studies, thereby improving evidence quality at its source. Specifically, future work should emphasize prospective sample size estimation and learning curve analysis during development, enhance model interpretability, conduct independent external validation, and ultimately assess the real-world impact of a model on clinical workflows and patient outcomes via pragmatic effectiveness studies. Additionally, we recommend using QUADAS-AI (Quality Assessment of Diagnostic Accuracy Studies Using AI) to assess the risk of bias in included original studies for diagnostic ML tasks [87]. QUADAS-AI focuses on data sources, sample size, eligibility criteria, the rationale for splitting training, validation, test sets, imaging protocols, and preprocessing methods—rather than simply labeling all case-control studies as “high risk.” In the studies we included, a substantial number of studies clearly described the source, size, and eligibility criteria, the principles of splitting into training, validation, and test sets, as well as the imaging protocols and preprocessing methods, thereby mitigating the high risk of bias associated with the study design.

This study has high statistical heterogeneity. Even when we conducted subgroup analyses using various ML approaches and validation methods to further elucidate the sources of heterogeneity, our explanatory power remains limited. This may be due to underlying heterogeneity present during model training. We consider that the following ML processes may introduce potential heterogeneity. First, regarding image preprocessing, the included studies exhibited certain variations in their approaches. These differences may introduce significant heterogeneity. For different images, there is considerable variation in image parameters, yet there is a lack of effective discussion on how this impacts modeling. The images in the original studies exhibit significant variations across imaging protocols. Although we performed subgroup analyses based on different models, even within the same model, differences in model iterations and parameter adjustment rules persist, potentially contributing to heterogeneity.

### Strengths and Limitations of the Study

Our systematic review is the first to quantitatively examine ML performance in LVH detection. While informative, several limitations should be considered when interpreting these results. First, the systematic search, while rigorous, identified solely small case-control studies, thereby narrowing the spectrum of studies for subgroup comparisons. Second, the limited number of included studies precluded meaningful comparisons between different ML methodologies. Third, while most studies completed external validation, some relied solely on random sampling for internal validation. Owing to the limited number of publications, a systematic comparison between internal and external validation approaches is infeasible. Fourth, the RoB in the included studies is high, which is inevitable given the stringent criteria for evaluating ML-based research. This observation corresponds with methodological appraisals indicating that 87% of ML models for medical prediction carry a high RoB [88]. Future studies should optimize design, implementation, reporting, and validation approaches to facilitate clinical translation of ML-based prediction models. Fifth, some studies divide data according to images, and patients’ images are shared between the training set and validation sets, which may result in data leakage. Sixth, there are different reference standards for different models. This discrepancy in reference standards indicates that any comparison of diagnostic accuracy between different model types is inherently confounded. The presented pooled estimates for each subgroup should be interpreted independently and not compared directly to each other. Seventh, the findings of this study may have been influenced to some extent by the fact that 3 papers used the 2×2 formula for estimation. The results should be interpreted with caution. Eighth, several included studies used “clinical consensus” as the reference standard for diagnosing LVH. While this consensus typically adhered to established guidelines (defining LVH as left ventricular mass index >115 g/m² in men and >95 g/m² in women [89]), original studies generally did not explicitly describe whether the consensus process was entirely independent of the outputs of evaluated ML models. Theoretically, “incorporation bias” could be introduced if the predictions of a model were known or incorporated during the adjudication of the reference standard, potentially leading to an overestimation of its diagnostic accuracy. This constitutes a key limitation for evidence interpretation in this systematic review and underscores the necessity for future original research to explicitly report reference standard independence in methodological descriptions.

### Future Directions

ML is widely used in LVH research, but its clinical application still faces multiple challenges. Many models are developed based on limited samples and lack standardized processes for key steps, such as image selection, segmentation, and feature extraction, resulting in high reliance of models on the experience of developers [62,90]. This raises concerns about the robustness of the models. Significant differences in image quality across different institutions further weaken the universality of the models. Existing models often focus on accuracy and neglect population risk stratification and regional differences in medical resources, limiting their clinical utility. Considering interpretability and a lack of trust among physicians, it remains difficult to integrate ML into clinical practice [91,92]. Although some models perform well in retrospective studies, their effectiveness and applicability in real-world clinical settings still need to be verified through rigorous prospective trials. Before clinical application, it is necessary to fully address the issues of patient data privacy protection, lack of trust among doctors due to insufficient model interpretability, and ethical considerations associated with new systems [93-95]. Additionally, we must be wary of overreliance on technological means at the expense of humanistic care. The need for systematic management and individualized treatment should not be overlooked. Technology should serve, rather than replace, patient-centered medical practices.

To promote the safe, effective, and responsible application of ML in LVH clinical practice, it is necessary to standardize the model development process, strengthen research on model generalizability and interpretability, conduct rigorous prospective validation, and focus on risk stratification and resource differences. Furthermore, it is necessary to establish a comprehensive ethical and regulatory framework covering data privacy, patient rights, algorithm transparency, and clinical workflow integration. Clear implementation guidelines are needed to ensure that technological development truly benefits patients and improves the quality of medical care.

### Conclusions

This study suggests that ML demonstrates diagnostic potential in detecting LVH. However, the overall certainty of the evidence is low, primarily constrained by the limited number of included studies and substantial heterogeneity. Consequently, current conclusions about model performance should be cautiously interpreted. Future investigations should adopt prospective designs and implement standardized data acquisition and model validation protocols to develop and evaluate ML models with enhanced robustness and clinical interpretability. This will advance this technology and make it a reliable diagnostic tool.

## Supplementary material

10.2196/76637 Multimedia Appendix 1 Literature search strategy.

10.2196/76637 Multimedia Appendix 2 Summary receiver operating characteristic curves, funnel plots, and nomograms for electrocardiogram (overall and validation analyses) and clinical features.

10.2196/76637 Checklist 1 PRISMA flowchart.

## Abbreviations

AI: artificial intelligence
DL: deep learning
DOR: diagnostic odds ratio
ECG: electrocardiogram
ECHO: echocardiography
EPV: events per variable
LVH: left ventricular hypertrophy
MeSH: Medical Subject Headings
ML: machine learning
NLR: negative likelihood ratio
PLR: positive likelihood ratio
PRISMA: Preferred Reporting Items for Systematic Reviews and Meta-Analyses
PROBAST: Prediction Model Risk of Bias Assessment Tool
QUADAS-AI: Quality Assessment of Diagnostic Accuracy Studies Using AI
RoB: risk of bias
SROC: summary receiver operating characteristic
SVM: support vector machine

## References

[1] Zhang X, He C, Lu S (2024). Construction and validation of a nomogram to predict left ventricular hypertrophy in low‐risk patients with hypertension. J Clin Hypertens.

[2] Đorđević DB, Koračević GP, Đorđević AD, Lović DB (2024). Hypertension and left ventricular hypertrophy. J Hypertens.

[3] Han Y, Li Y, Wu Z (2024). Progress in diagnosis and treatment of hypertension combined with left ventricular hypertrophy. Ann Med.

[4] Miller RJH, Mikami Y, Heydari B (2020). Sex-specific relationships between patterns of ventricular remodelling and clinical outcomes. Eur Heart J Cardiovasc Imaging.

[5] Lewis AA, Ayers CR, Selvin E (2020). Racial differences in malignant left ventricular hypertrophy and incidence of heart failure: a multicohort study. Circulation.

[6] Cao X, Broughton ST, Waits GS, Nguyen T, Li Y, Soliman EZ (2019). Interrelations between hypertension and electrocardiographic left ventricular hypertrophy and their associations with cardiovascular mortality. Am J Cardiol.

[7] Siranart N, Deepan N, Techasatian W (2024). Diagnostic accuracy of artificial intelligence in detecting left ventricular hypertrophy by electrocardiograph: a systematic review and meta-analysis. Sci Rep.

[8] Pedersen LR, Kristensen AMD, Petersen SS (2020). Prognostic implications of left ventricular hypertrophy diagnosed on electrocardiogram vs echocardiography. J Clin Hypertens (Greenwich).

[9] Woythaler JN, Singer SL, Kwan OL (1983). Accuracy of echocardiography versus electrocardiography in detecting left ventricular hypertrophy: comparison with postmortem mass measurements. J Am Coll Cardiol.

[10] Cai C, Imai T, Hasumi E, Fujiu K (2024). One-shot screening: utilization of a two-dimensional convolutional neural network for automatic detection of left ventricular hypertrophy using electrocardiograms. Comput Methods Programs Biomed.

[11] Spahillari A, McCormick I, Yang JX, Quinn GR, Manning WJ (2019). On-call transthoracic echocardiographic interpretation by first year cardiology fellows: comparison with attending cardiologists. BMC Med Educ.

[12] Rao S, Ferris TG, Hidrue MK (2020). Physician burnout, engagement and career satisfaction in a large academic medical practice. Clin Med Res.

[13] Quinn GR, Ranum D, Song E (2017). Missed diagnosis of cardiovascular disease in outpatient general medicine: insights from malpractice claims data. Jt Comm J Qual Patient Saf.

[14] Wang Z, Gu Y, Huang L (2024). Construction of machine learning diagnostic models for cardiovascular pan-disease based on blood routine and biochemical detection data. Cardiovasc Diabetol.

[15] Qi X, Wang S, Fang C, Jia J, Lin L, Yuan T (2025). Machine learning and SHAP value interpretation for predicting comorbidity of cardiovascular disease and cancer with dietary antioxidants. Redox Biol.

[16] Layton AT (2024). AI, machine learning, and ChatGPT in hypertension. Hypertension.

[17] Ying Y, Ju R, Wang J (2025). Accuracy of machine learning in diagnosing microsatellite instability in gastric cancer: a systematic review and meta-analysis. Int J Med Inform.

[18] Beam AL, Kohane IS (2018). Big data and machine learning in health care. J Am Med Assoc.

[19] Zhao K, Zhu Y, Chen X (2024). Machine learning in hypertrophic cardiomyopathy: nonlinear model from clinical and CMR features predicting cardiovascular events. JACC Cardiovasc Imaging.

[20] You J, Guo Y, Kang JJ (2023). Development of machine learning-based models to predict 10-year risk of cardiovascular disease: a prospective cohort study. Stroke Vasc Neurol.

[21] Naderi H, Ramírez J, van Duijvenboden S (2024). Diagnostic and prognostic value of ECG-predicted hypertension-mediated left ventricular hypertrophy using machine learning. medRxiv.

[22] Ryu JS, Lee S, Chu Y, Ahn MS, Park YJ, Yang S (2023). CoAt-mixer: self-attention deep learning framework for left ventricular hypertrophy using electrocardiography. PLoS ONE.

[23] Page MJ, McKenzie JE, Bossuyt PM (2021). The PRISMA 2020 statement: an updated guideline for reporting systematic reviews. BMJ.

[24] Moons KGM, Wolff RF, Riley RD (2019). PROBAST: a tool to assess risk of bias and applicability of prediction model studies: explanation and elaboration. Ann Intern Med.

[25] Deeks JJ, Macaskill P, Irwig L (2005). The performance of tests of publication bias and other sample size effects in systematic reviews of diagnostic test accuracy was assessed. J Clin Epidemiol.

[26] Fagan TJ (1975). Letter: nomogram for Bayes’s theorem. N Engl J Med.

[27] Wan J, Wang P, Liu S, Wang X, Zhou P, Yang J (2024). Risk factors and a predictive model for left ventricular hypertrophy in young adults with salt-sensitive hypertension. J Clin Hypertens (Greenwich).

[28] Farhad M, Masud MM, Beg A, Ahmad A, Ahmed LA, Memon S (2023). A data-efficient zero-shot and few-shot Siamese approach for automated diagnosis of left ventricular hypertrophy. Comput Biol Med.

[29] Yuan R, Chen J, Zhang S, Zhang X, Yu J (2023). Establishment of an individual-specific nomogram for predicting the risk of left ventricular hypertrophy in Chinese postmenopausal hypertensive women. Medicina (Kaunas).

[30] Dwivedi T, Xue J, Treiman D, Dubey A, Albert D (2023). Machine learning models of 6-lead ECGs for the interpretation of left ventricular hypertrophy (LVH). J Electrocardiol.

[31] Liu CW, Wu FH, Hu YL (2023). Left ventricular hypertrophy detection using electrocardiographic signal. Sci Rep.

[32] Zhao X, Huang G, Wu L (2022). Deep learning assessment of left ventricular hypertrophy based on electrocardiogram. Front Cardiovasc Med.

[33] Liu CM, Hsieh ME, Hu YF (2022). Artificial intelligence-enabled model for early detection of left ventricular hypertrophy and mortality prediction in young to middle-aged adults. Circ Cardiovasc Qual Outcomes.

[34] Wu Z, Shi M, Wang L, Yao Y (2022). Identification of major risk factors and non-linear effects to the development of left ventricular hypertrophy in chronic kidney disease by constructing and validation of nomograms. Front Med (Lausanne).

[35] Yu X, Yao X, Wu B (2022). Using deep learning method to identify left ventricular hypertrophy on echocardiography. Int J Cardiovasc Imaging.

[36] Kokubo T, Kodera S, Sawano S (2022). Automatic detection of left ventricular dilatation and hypertrophy from electrocardiograms using deep learning. Int Heart J.

[37] Ye C, Wang T, Gong J (2021). Development of a nomogram for screening the risk of left ventricular hypertrophy in Chinese hypertensive patients. J Clin Hyperten.

[38] Angelaki E, Marketou ME, Barmparis GD (2021). Detection of abnormal left ventricular geometry in patients without cardiovascular disease through machine learning: an ECG-based approach. J Clin Hypertens (Greenwich).

[39] Ghorbani A, Ouyang D, Abid A (2020). Deep learning interpretation of echocardiograms. NPJ Digit Med.

[40] Kwon JM, Jeon KH, Kim HM (2020). Comparing the performance of artificial intelligence and conventional diagnosis criteria for detecting left ventricular hypertrophy using electrocardiography. Europace.

[41] Lin GM, Liu K (2020). An electrocardiographic system with anthropometrics via machine learning to screen left ventricular hypertrophy among young adults. IEEE J Transl Eng Health Med.

[42] Tison GH, Zhang J, Delling FN, Deo RC (2019). Automated and interpretable patient ECG profiles for disease detection, tracking, and discovery. Circ Cardiovasc Qual Outcomes.

[43] Sparapani R, Dabbouseh NM, Gutterman D (2019). Detection of left ventricular hypertrophy using Bayesian additive regression trees: the MESA. J Am Heart Assoc.

[44] Madani A, Ong JR, Tibrewal A, Mofrad MRK (2018). Deep echocardiography: data-efficient supervised and semi-supervised deep learning towards automated diagnosis of cardiac disease. NPJ Digit Med.

[45] De la Garza Salazar F, Romero Ibarguengoitia ME, Azpiri López JR, González Cantú A (2021). Optimizing ECG to detect echocardiographic left ventricular hypertrophy with computer-based ECG data and machine learning. PLoS ONE.

[46] Huang JT, Tseng CH, Huang WM (2025). Comparison of machine learning and conventional criteria in detecting left ventricular hypertrophy and prognosis with electrocardiography. Eur Heart J Digit Health.

[47] Taconne M, Corino VDA, Mainardi L (2025). An ECG-based model for left ventricular hypertrophy detection: a machine learning approach. IEEE Open J Eng Med Biol.

[48] van Smeden M, Moons KG, de Groot JA (2019). Sample size for binary logistic prediction models: beyond events per variable criteria. Stat Methods Med Res.

[49] Duffy G, Cheng PP, Yuan N (2022). High-throughput precision phenotyping of left ventricular hypertrophy with cardiovascular deep learning. JAMA Cardiol.

[50] Liu LS, Writing Group of 2010 Chinese Guidelines for the Management of Hypertension (2011). 2010 Chinese guidelines for the management of hypertension. Zhonghua Xin Xue Guan Bing Za Zhi.

[51] Casale PN, Devereux RB, Kligfield P (1985). Electrocardiographic detection of left ventricular hypertrophy: development and prospective validation of improved criteria. J Am Coll Cardiol.

[52] Sokolow M, Lyon TP (1949). The ventricular complex in left ventricular hypertrophy as obtained by unipolar precordial and limb leads. Am Heart J.

[53] Xia Y, Li X, Zhang H (2020). Diagnostic capability and influence factors for a new electrocardiogram criterion in the diagnosis of left ventricular hypertrophy in a Chinese population. Cardiology.

[54] Wang D, Xu JZ, Zhang W (2020). Performance of electrocardiographic criteria for echocardiographically diagnosed left ventricular hypertrophy in Chinese hypertensive patients. Am J Hypertens.

[55] Bang CN, Devereux RB, Okin PM (2014). Regression of electrocardiographic left ventricular hypertrophy or strain is associated with lower incidence of cardiovascular morbidity and mortality in hypertensive patients independent of blood pressure reduction—a LIFE review. J Electrocardiol.

[56] Hancock EW, Deal BJ, Mirvis DM (2009). AHA/ACCF/HRS recommendations for the standardization and interpretation of the electrocardiogram: part V: electrocardiogram changes associated with cardiac chamber hypertrophy: a scientific statement from the American Heart Association Electrocardiography and Arrhythmias Committee, Council on Clinical Cardiology; the American College of Cardiology Foundation; and the Heart Rhythm Society: endorsed by the International Society for Computerized Electrocardiology. Circulation.

[57] Rautaharju PM, Soliman EZ (2014). Electrocardiographic left ventricular hypertrophy and the risk of adverse cardiovascular events: a critical appraisal. J Electrocardiol.

[58] Jiang Y, Yang M, Wang S, Li X, Sun Y (2020). Emerging role of deep learning-based artificial intelligence in tumor pathology. Cancer Commun (Lond).

[59] Jiang X, Li J, Kan Y (2021). MRI based radiomics approach with deep learning for prediction of vessel invasion in early-stage cervical cancer. IEEE/ACM Trans Comput Biol Bioinform.

[60] She L, Li Y, Wang H (2025). Imaging-based AI for predicting lymphovascular space invasion in cervical cancer: systematic review and meta-analysis. J Med Internet Res.

[61] Zuo H, Huang B, He J, Fang L, Huang M (2025). Machine learning approaches in high myopia: systematic review and meta-analysis. J Med Internet Res.

[62] Zhang H, Zou P, Luo P, Jiang X (2025). Machine learning for the early prediction of delayed cerebral ischemia in patients with subarachnoid hemorrhage: systematic review and meta-analysis. J Med Internet Res.

[63] Zhu J, Yang F, Wang Y (2024). Accuracy of machine learning in discriminating Kawasaki disease and other febrile illnesses: systematic review and meta-analysis. J Med Internet Res.

[64] Becker T, Rousseau AJ, Geubbelmans M, Burzykowski T, Valkenborg D (2023). Decision trees and random forests. Am J Orthod Dentofacial Orthop.

[65] Liu L, Li Z, Hu Y (2025). Predictive performance of machine learning for suicide in adolescents: systematic review and meta-analysis. J Med Internet Res.

[66] Yang J, Zeng S, Cui S, Zheng J, Wang H (2025). Predictive modeling of acute respiratory distress syndrome using machine learning: systematic review and meta-analysis. J Med Internet Res.

[67] Holzinger A, Langs G, Denk H, Zatloukal K, Müller H (2019). Causability and explainability of artificial intelligence in medicine. Wiley Interdiscip Rev Data Min Knowl Discov.

[68] Ngiam KY, Khor IW (2019). Big data and machine learning algorithms for health-care delivery. Lancet Oncol.

[69] Parvatikar PP, Patil S, Khaparkhuntikar K (2023). Artificial intelligence: machine learning approach for screening large database and drug discovery. Antiviral Res.

[70] Tsai CA, Chang YJ (2023). Efficient selection of Gaussian kernel SVM parameters for imbalanced data. Genes (Basel).

[71] Park JS, Park SY, Moon JW, Kim K, Suh DI (2025). Artificial intelligence models for pediatric lung sound analysis: systematic review and meta-analysis. J Med Internet Res.

[72] Liao YY, Gao K, Fu BW (2021). Risk factors for electrocardiographic left ventricular hypertrophy in a young Chinese general population: the Hanzhong adolescent cohort study. BMC Cardiovasc Disord.

[73] Tiyerili V, Müller CFH, Fung S, Panek D, Nickenig G, Becher UM (2012). Estrogen improves vascular function via peroxisome-proliferator-activated-receptor-γ. J Mol Cell Cardiol.

[74] Vasan RS, Song RJ, Xanthakis V (2022). Hypertension-mediated organ damage: prevalence, correlates, and prognosis in the community. Hypertension.

[75] Wang C, Yuan Y, Zheng M (2020). Association of age of onset of hypertension with cardiovascular diseases and mortality. J Am Coll Cardiol.

[76] Zhou B, Li C, Shou J, Zhang Y, Wen C, Zeng C (2020). The cumulative blood pressure load and target organ damage in patients with essential hypertension. J Clin Hypertens (Greenwich).

[77] Carlsson AC, Ruge T, Sundström J (2013). Association between circulating endostatin, hypertension duration, and hypertensive target-organ damage. Hypertension.

[78] Zhang X, Li G, Zhang D, Sun Y (2024). Influence of hypertension and global or abdominal obesity on left ventricular hypertrophy: a cross-sectional study. J Clin Hypertens (Greenwich).

[79] Tsujimoto T, Kajio H (2017). Abdominal obesity is associated with an increased risk of all-cause mortality in patients with HFpEF. J Am Coll Cardiol.

[80] Choi D, Choi S, Son JS, Oh SW, Park SM (2019). Impact of discrepancies in general and abdominal obesity on major adverse cardiac events. J Am Heart Assoc.

[81] Ross R, Neeland IJ, Yamashita S (2020). Waist circumference as a vital sign in clinical practice: a Consensus Statement from the IAS and ICCR Working Group on Visceral Obesity. Nat Rev Endocrinol.

[82] Wang Y, Howard AG, Adair LS, Wang H, Avery CL, Gordon-Larsen P (2020). Waist circumference change is associated with blood pressure change independent of BMI change. Obesity (Silver Spring).

[83] Wu D, Ono R, Wang S, Kobayashi Y, Sughimoto K, Liu H (2024). Pulse wave signal-driven machine learning for identifying left ventricular enlargement in heart failure patients. Biomed Eng Online.

[84] Lau ES, Di Achille P, Kopparapu K (2023). Deep learning-enabled assessment of left heart structure and function predicts cardiovascular outcomes. J Am Coll Cardiol.

[85] Wehbe RM, Katsaggelos AK, Hammond KJ (2023). Deep learning for cardiovascular imaging: a review. JAMA Cardiol.

[86] Maleki F, Ovens K, Gupta R, Reinhold C, Spatz A, Forghani R (2023). Generalizability of machine learning models: quantitative evaluation of three methodological pitfalls. Radiol Artif Intell.

[87] Sounderajah V, Ashrafian H, Rose S (2021). A quality assessment tool for artificial intelligence-centered diagnostic test accuracy studies: QUADAS-AI. Nat Med.

[88] Andaur Navarro CL, Damen JAA, Takada T (2021). Risk of bias in studies on prediction models developed using supervised machine learning techniques: systematic review. BMJ.

[89] Lang RM, Badano LP, Mor-Avi V (2015). Recommendations for cardiac chamber quantification by echocardiography in adults: an update from the American Society of Echocardiography and the European Association of Cardiovascular Imaging. J Am Soc Echocardiogr.

[90] Wang B, Jiang B, Liu D, Zhu R (2025). Early predictive accuracy of machine learning for hemorrhagic transformation in acute ischemic stroke: systematic review and meta-analysis. J Med Internet Res.

[91] Kelly CJ, Karthikesalingam A, Suleyman M, Corrado G, King D (2019). Key challenges for delivering clinical impact with artificial intelligence. BMC Med.

[92] Alkhanbouli R, Matar Abdulla Almadhaani H, Alhosani F, Simsekler MCE (2025). The role of explainable artificial intelligence in disease prediction: a systematic literature review and future research directions. BMC Med Inform Decis Mak.

[93] Aggarwal R, Farag S, Martin G, Ashrafian H, Darzi A (2021). Patient perceptions on data sharing and applying artificial intelligence to health care data: cross-sectional survey. J Med Internet Res.

[94] Murdoch B (2021). Privacy and artificial intelligence: challenges for protecting health information in a new era. BMC Med Ethics.

[95] Larson DB, Magnus DC, Lungren MP, Shah NH, Langlotz CP (2020). Ethics of using and sharing clinical imaging data for artificial intelligence: a proposed framework. Radiology.

