# Supplementary material for: Machine Learning in Left Ventricular Hypertrophy Detection: Systematic Review and Meta-Analysis
Source: J Med Internet Res. 2026 Feb 27;28:e76637. doi: 10.2196/76637 (PMC12954682; doi:10.2196/76637)
Supplement: Multimedia Appendix 1 [file jmir-v28-e76637-s001.docx]

# **Table S1 Literature search strategy**

Search date: November 12, 2025

Databases searched: PubMed, Cochrane Library, EMBASE, and Web of Science.

**Search Strategy in PUBMED**

| Search number | Query | Results |
| --- | --- | --- |
| #1 | "Hypertrophy, Left Ventricular"[Mesh] | 15,835 |
| #2 | ((((((((((((Hypertrophy, Left Ventricular[Title/Abstract]) OR (Left Ventricular Hypertrophy[Title/Abstract])) OR (Left Ventricular Hypertrophies[Title/Abstract])) OR (cor bovinum[Title/Abstract])) OR (heart left ventricle hypertrophy[Title/Abstract])) OR (left cardiac ventricle hypertrophy[Title/Abstract])) OR (left cardiac ventricular hypertrophy[Title/Abstract])) OR (left heart ventricle concentric hypertrophy[Title/Abstract])) OR (left heart ventricle hypertrophy[Title/Abstract])) OR (left heart ventricular hypertrophy[Title/Abstract])) OR (left ventricular concentric hypertrophy[Title/Abstract])) OR (LV hypertrophies[Title/Abstract])) OR (LV hypertrophy[Title/Abstract]) | 22,855 |
| #3 | ("Hypertrophy, Left Ventricular"[Mesh]) OR ((((((((((((Hypertrophy, Left Ventricular[Title/Abstract]) OR (Left Ventricular Hypertrophy[Title/Abstract])) OR (Left Ventricular Hypertrophies[Title/Abstract])) OR (cor bovinum[Title/Abstract])) OR (heart left ventricle hypertrophy[Title/Abstract])) OR (left cardiac ventricle hypertrophy[Title/Abstract])) OR (left cardiac ventricular hypertrophy[Title/Abstract])) OR (left heart ventricle concentric hypertrophy[Title/Abstract])) OR (left heart ventricle hypertrophy[Title/Abstract])) OR (left heart ventricular hypertrophy[Title/Abstract])) OR (left ventricular concentric hypertrophy[Title/Abstract])) OR (LV hypertrophies[Title/Abstract])) OR (LV hypertrophy[Title/Abstract]) | 28,964 |
| #4 | machine learning[MeSH Terms] | 106,802 |
| #5 | ((((((((((((((((((((((((((Machine Learning[Title/Abstract]) OR (Transfer Learning[Title/Abstract])) OR (Deep learning[Title/Abstract])) OR (Ensemble Learning[Title/Abstract])) OR (artificial intelligence[Title/Abstract])) OR (random forest[Title/Abstract])) OR (neural network[Title/Abstract])) OR (neural networks[Title/Abstract])) OR (K-Nearest Neighbor[Title/Abstract])) OR (Support vector machine[Title/Abstract])) OR (SVM[Title/Abstract])) OR (Gradient Boosting Machine[Title/Abstract])) OR (Nomogram[Title/Abstract])) OR (XGBoost[Title/Abstract])) OR (Adaboost[Title/Abstract])) OR (Decision tree[Title/Abstract])) OR (Naive Bayesian[Title/Abstract])) OR (Multilayer perceptron[Title/Abstract])) OR (Bayesian network[Title/Abstract])) OR (Radiomics[Title/Abstract])) OR (Radiomic[Title/Abstract])) OR (Prediction model[Title/Abstract])) OR (CNN[Title/Abstract])) OR (AlexNet[Title/Abstract])) OR (VGGNet[Title/Abstract])) OR (ResNet[Title/Abstract])) OR (GoogLeNet[Title/Abstract]) | 486,237 |
| #6 | (machine learning[MeSH Terms]) OR (((((((((((((((((((((((((((machine learning[Title/Abstract]) OR (Transfer Learning[Title/Abstract])) OR (Deep learning[Title/Abstract])) OR (Ensemble Learning[Title/Abstract])) OR (artificial intelligence[Title/Abstract])) OR (random forest[Title/Abstract])) OR (neural network[Title/Abstract])) OR (neural networks[Title/Abstract])) OR (K-Nearest Neighbor[Title/Abstract])) OR (Support vector machine[Title/Abstract])) OR (SVM[Title/Abstract])) OR (Gradient Boosting Machine[Title/Abstract])) OR (Nomogram[Title/Abstract])) OR (XGBoost[Title/Abstract])) OR (Adaboost[Title/Abstract])) OR (Decision tree[Title/Abstract])) OR (Naive Bayesian[Title/Abstract])) OR (Multilayer perceptron[Title/Abstract])) OR (Bayesian network[Title/Abstract])) OR (Radiomics[Title/Abstract])) OR (Radiomic[Title/Abstract])) OR (Prediction model[Title/Abstract])) OR (CNN[Title/Abstract])) OR (AlexNet[Title/Abstract])) OR (VGGNet[Title/Abstract])) OR (ResNet[Title/Abstract])) OR (GoogLeNet[Title/Abstract])) | 493,901 |
| #7 | #3 AND #6 | 229 |

**Search Strategy in Cochrane Library**

| Search number | Query | Results |
| --- | --- | --- |
| #1 | MeSH descriptor: [Hypertrophy, Left Ventricular] explode all trees | 1,118 |
| #2 | (Hypertrophy, Left Ventricular):ti,ab,kw OR (Left Ventricular Hypertrophy):ti,ab,kw OR (Left Ventricular Hypertrophies):ti,ab,kw OR (cor bovinum):ti,ab,kw OR (heart left ventricle hypertrophy):ti,ab,kw | 2,718 |
| #3 | (left cardiac ventricle hypertrophy):ti,ab,kw OR (left cardiac ventricular hypertrophy):ti,ab,kw OR (left heart ventricle concentric hypertrophy):ti,ab,kw OR (left heart ventricle hypertrophy):ti,ab,kw OR (left heart ventricular hypertrophy):ti,ab,kw | 2,076 |
| #4 | (left ventricular concentric hypertrophy):ti,ab,kw OR (LV hypertrophies):ti,ab,kw OR (LV hypertrophy):ti,ab,kw | 766 |
| #5 | #1 OR #2 OR #3 OR #4 | 2,764 |
| #6 | MeSH descriptor: [Machine Learning] explode all trees | 1,222 |
| #7 | (Machine Learning):ti,ab,kw OR (Transfer Learning):ti,ab,kw OR (Deep learning):ti,ab,kw OR (Ensemble Learning):ti,ab,kw OR (artificial intelligence):ti,ab,kw | 9,575 |
| #8 | (random forest):ti,ab,kw OR (neural network):ti,ab,kw OR (neural networks):ti,ab,kw OR (K-Nearest Neighbor):ti,ab,kw OR (Support vector machine):ti,ab,kw | 5,544 |
| #9 | (SVM):ti,ab,kw OR (Gradient Boosting Machine):ti,ab,kw OR (Nomogram):ti,ab,kw OR (XGBoost):ti,ab,kw OR (Adaboost):ti,ab,kw | 2,553 |
| #10 | (Decision tree):ti,ab,kw OR (Naive Bayesian):ti,ab,kw OR (Multilayer perceptron):ti,ab,kw OR (Bayesian network):ti,ab,kw OR (Radiomics):ti,ab,kw | 2,360 |
| #11 | (Radiomic):ti,ab,kw OR (Prediction model):ti,ab,kw OR (CNN):ti,ab,kw OR (AlexNet):ti,ab,kw OR (VGGNet):ti,ab,kw | 7,356 |
| #12 | (ResNet):ti,ab,kw OR (GoogLeNet):ti,ab,kw | 71 |
| #13 | #6 OR #7 OR #8 OR #9 OR #10 OR #11 OR #12 | 21,498 |
| #14 | #5 AND #13 | 37 |

**Search Strategy in EMBASE**

| Search number | Query | Results |
| --- | --- | --- |
| #1 | 'left ventricular hypertrophy'/exp | 48,898 |
| #2 | 'left ventricular hypertrophy':ab,ti OR 'hypertrophy, left ventricular':ab,ti OR 'left ventricular hypertrophies':ab,ti OR 'cor bovinum':ab,ti OR 'heart left ventricle hypertrophy':ab,ti OR 'left cardiac ventricle hypertrophy':ab,ti OR 'left cardiac ventricular hypertrophy':ab,ti OR 'left heart ventricle concentric hypertrophy':ab,ti OR 'left heart ventricle hypertrophy':ab,ti OR 'left heart ventricular hypertrophy':ab,ti OR 'left ventricular concentric hypertrophy':ab,ti OR 'lv hypertrophies':ab,ti OR 'lv hypertrophy':ab,ti | 36,285 |
| #3 | #1 OR #2 | 57,736 |
| #4 | 'machine learning'/exp | 659,594 |
| #5 | 'machine learning':ab,ti OR 'transfer learning':ab,ti OR 'deep learning':ab,ti OR 'ensemble learning':ab,ti OR 'artificial intelligence':ab,ti OR 'random forest':ab,ti OR 'neural network':ab,ti OR 'neural networks':ab,ti OR 'k-nearest neighbor':ab,ti OR 'support vector machine':ab,ti OR svm:ab,ti OR 'gradient boosting machine':ab,ti OR nomogram:ab,ti OR xgboost:ab,ti OR adaboost:ab,ti OR 'decision tree':ab,ti OR 'naive bayesian':ab,ti OR 'multilayer perceptron':ab,ti OR 'bayesian network':ab,ti OR radiomics:ab,ti OR 'prediction model':ab,ti OR cnn:ab,ti OR alexnet:ab,ti OR vggnet:ab,ti OR resnet:ab,ti OR googlenet:ab,ti | 550,862 |
| #6 | #4 OR #5 | 860,790 |
| #7 | #3 AND #6 | 615 |

**Search Strategy in Web of Science**

| Search number | Query | Results |
| --- | --- | --- |
| #1 | Hypertrophy, Left Ventricular (Topic) OR Left Ventricular Hypertrophy (Topic) OR Left Ventricular Hypertrophies (Topic) OR cor bovinum (Topic) OR heart left ventricle hypertrophy (Topic) OR left cardiac ventricle hypertrophy (Topic) OR left cardiac ventricular hypertrophy (Topic) OR left heart ventricle concentric hypertrophy (Topic) OR left heart ventricle hypertrophy (Topic) OR left heart ventricular hypertrophy (Topic) OR left ventricular concentric hypertrophy (Topic) OR LV hypertrophies (Topic) OR LV hypertrophy (Topic) | 22,247 |
| #2 | Machine Learning (Topic) OR Transfer Learning (Topic) OR Deep learning (Topic) OR Ensemble Learning (Topic) OR artificial intelligence (Topic) OR random forest (Topic) OR neural network (Topic) OR neural networks (Topic) OR K-Nearest Neighbor (Topic) OR Support vector machine (Topic) OR SVM (Topic) OR Gradient Boosting Machine (Topic) OR Nomogram (Topic) OR XGBoost (Topic) OR Adaboost (Topic) OR Decision tree (Topic) OR Naive Bayesian (Topic) OR Multilayer perceptron (Topic) OR Bayesian network (Topic) OR Radiomics (Topic) OR Radiomic (Topic) OR Prediction model (Topic) OR CNN (Topic) OR AlexNet (Topic) OR VGGNet (Topic) OR ResNet (Topic) OR GoogLeNet (Topic) | 1,794,858 |
| #3 | #1 AND #2 | 501 |
