# Supplementary material for: Machine Learning in Left Ventricular Hypertrophy Detection: Systematic Review and Meta-Analysis
Source: J Med Internet Res. 2026 Feb 27;28:e76637. doi: 10.2196/76637 (PMC12954682; doi:10.2196/76637)
Supplement: Multimedia Appendix 2 [file jmir-v28-e76637-s002.docx]

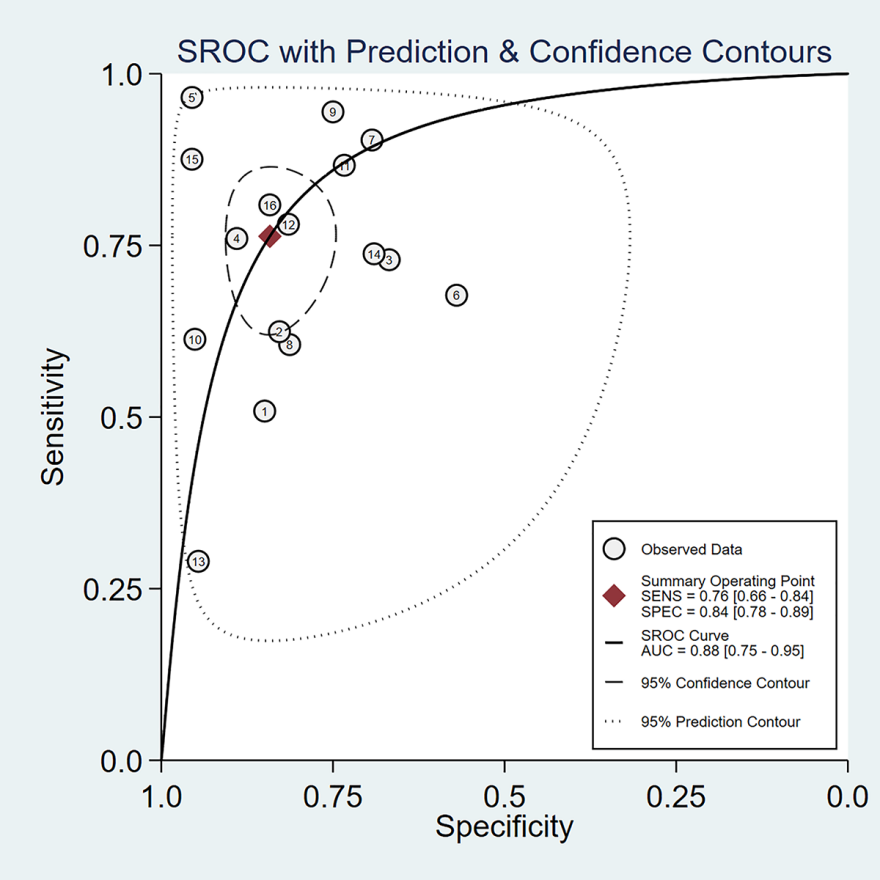


Figure S1 SROC of electrocardiogram


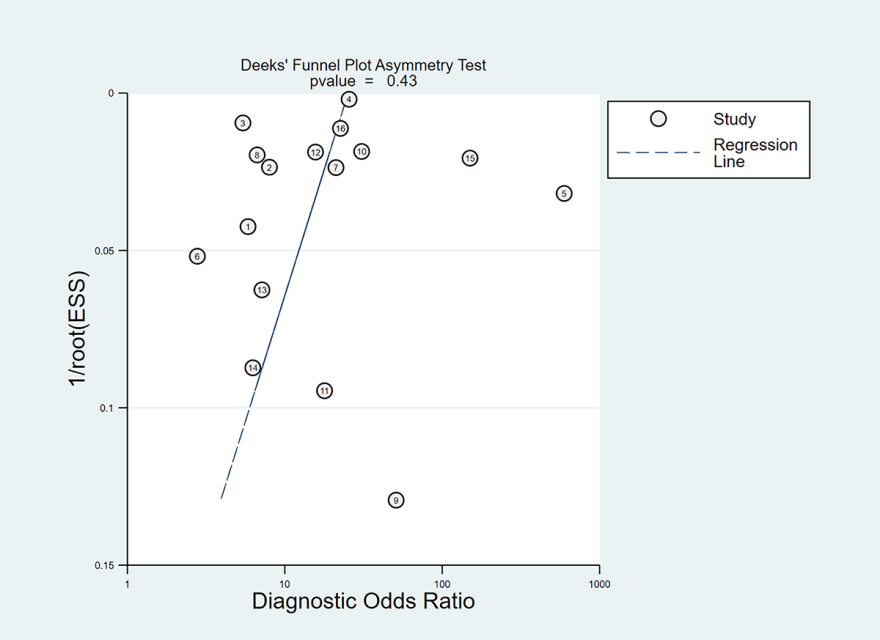


Figure S2 Funnel plot of electrocardiogram


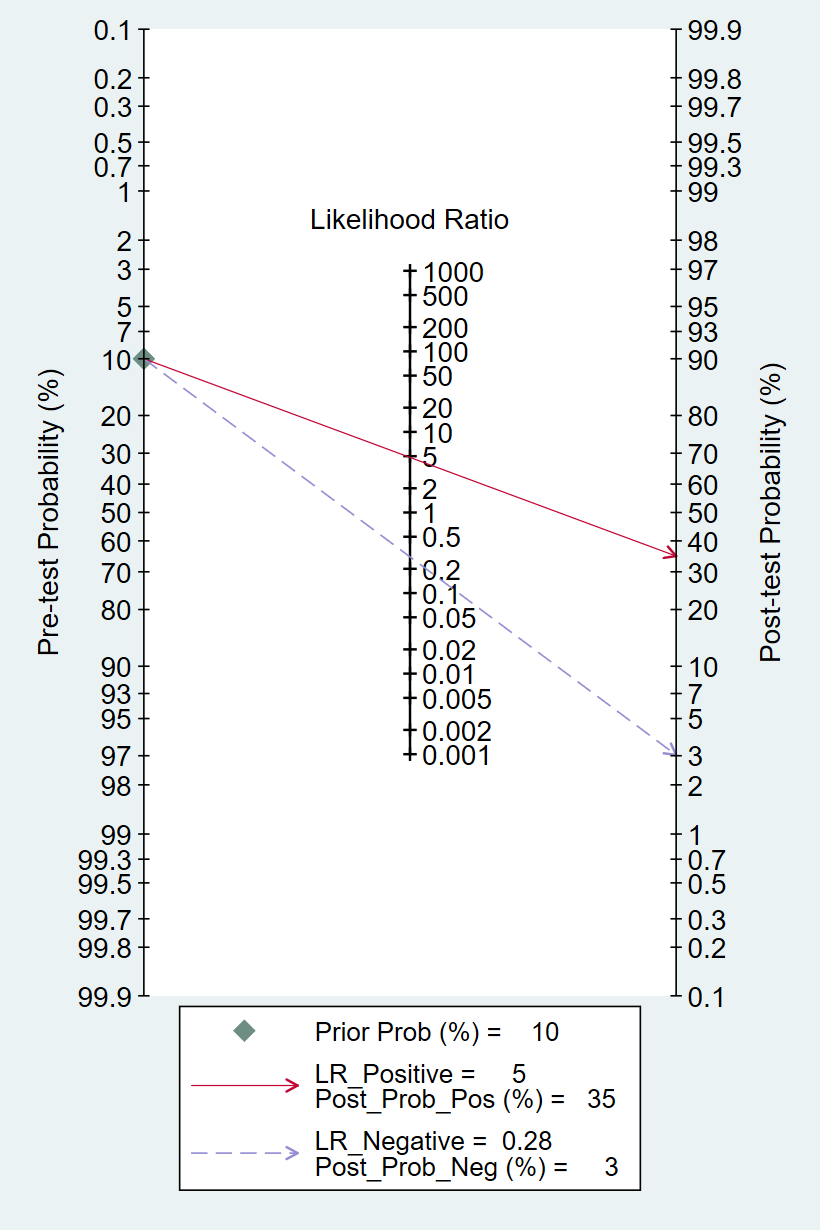


Figure S3 Nomogram of electrocardiogram


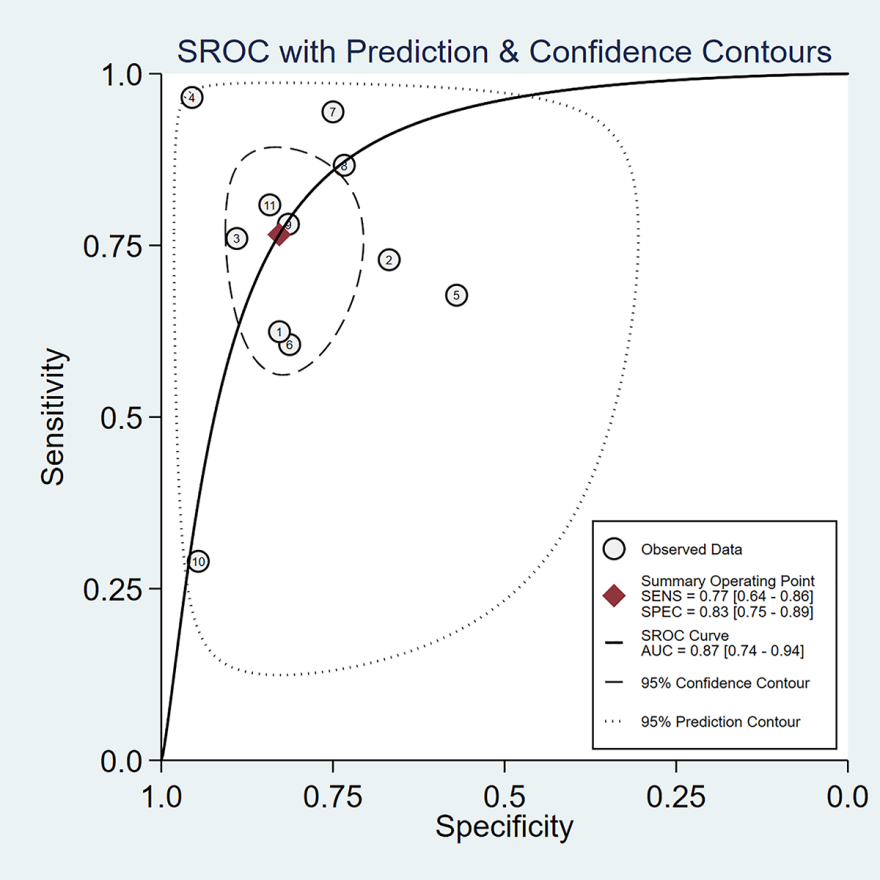


Figure S4 Subgroup analyses of electrocardiogram SROC for internal validation


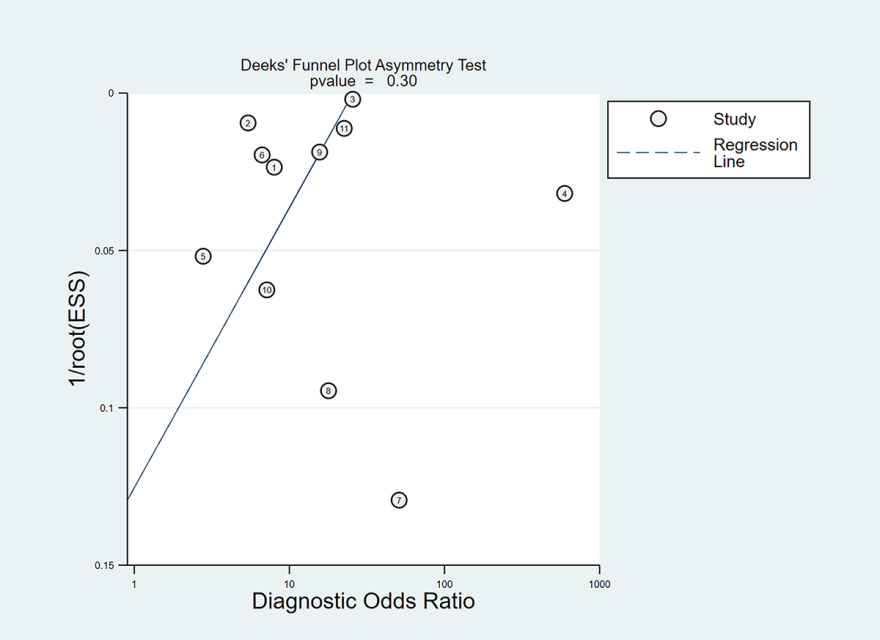


Figure S5 Subgroup analyses of electrocardiogram funnel plot for internal validation


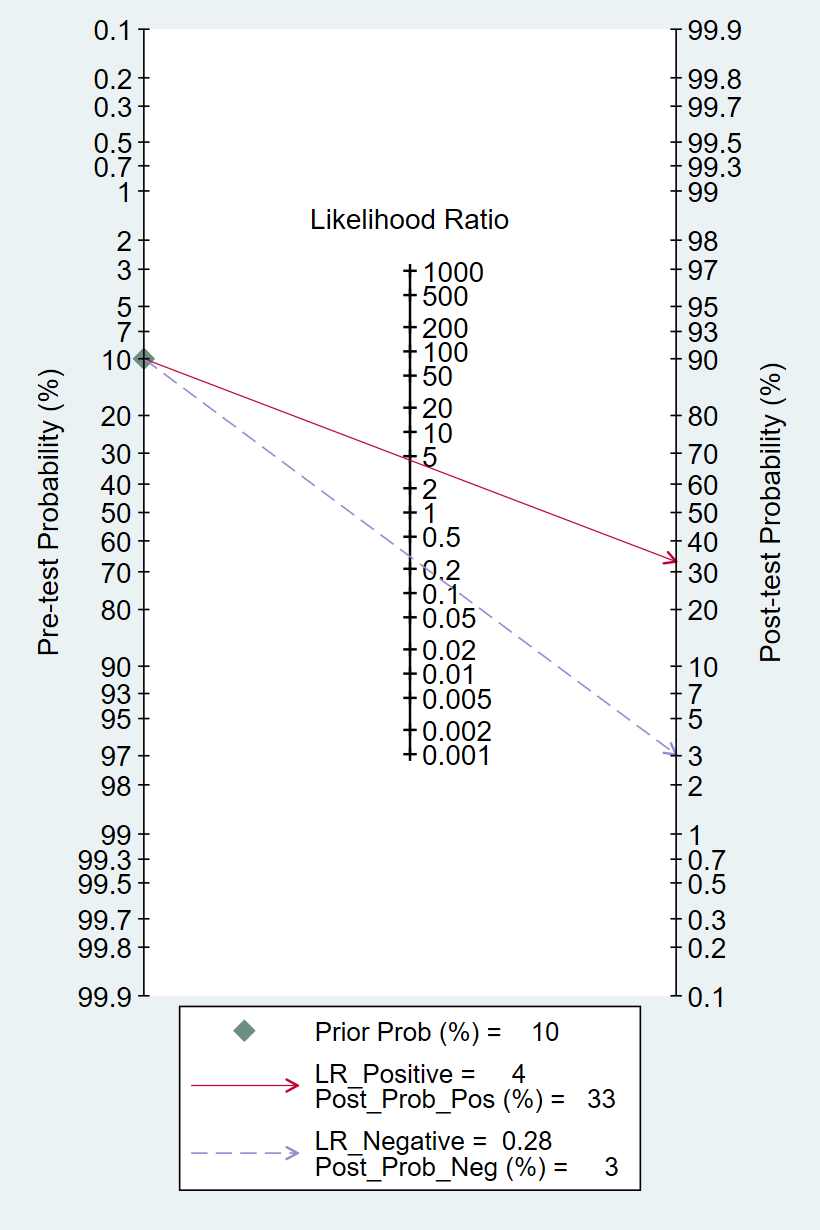


Figure S6 Subgroup analyses of electrocardiogram nomogram for internal validation


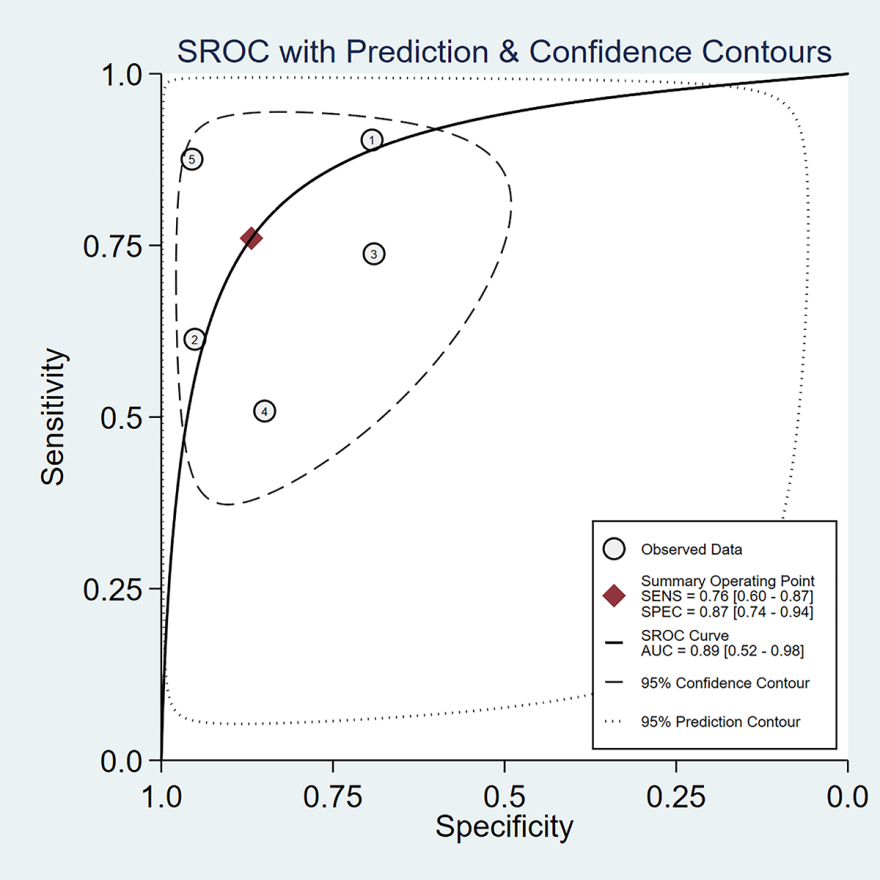


Figure S7 Subgroup analyses of electrocardiogram SROC for external validation


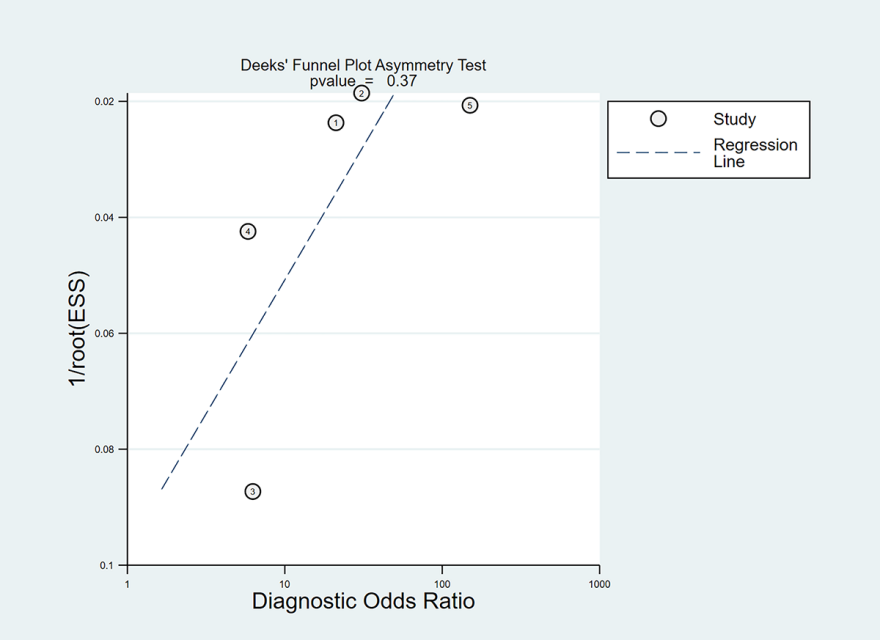


Figure S8 Subgroup analyses of electrocardiogram funnel plot for external validation


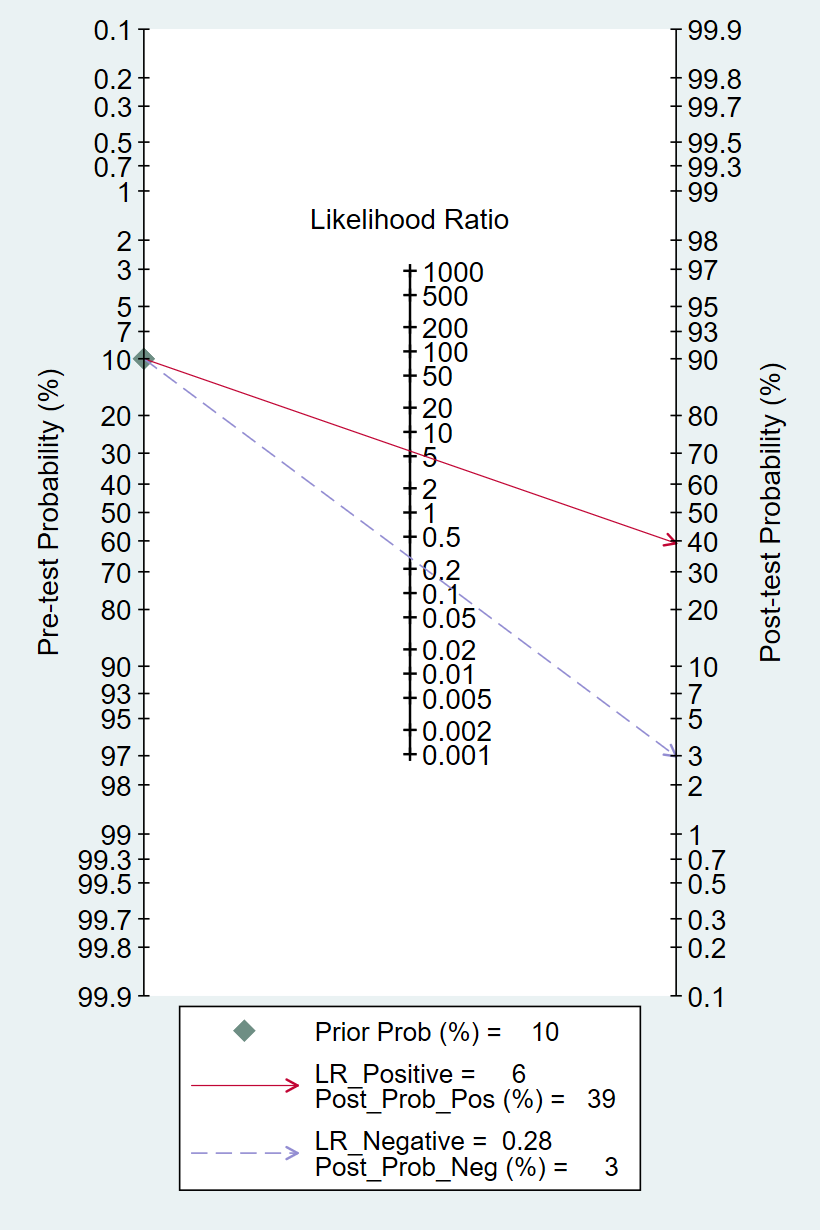


Figure S9 Subgroup analyses of electrocardiogram nomogram for external validation


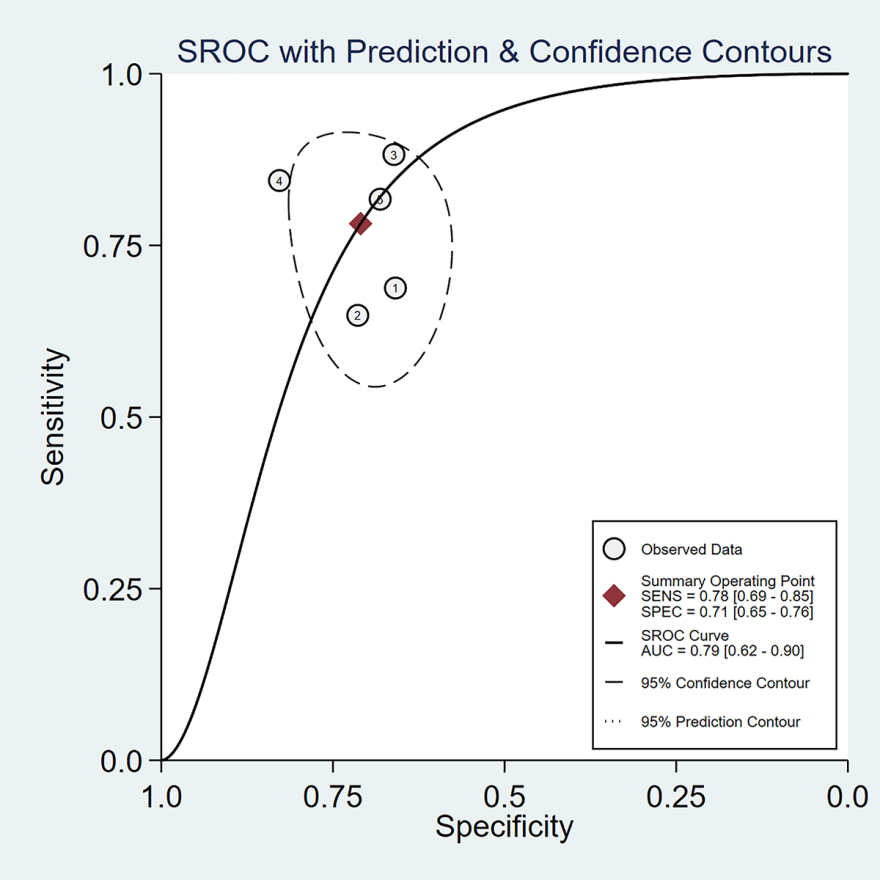


Figure S10 Clinical characterization of SROC


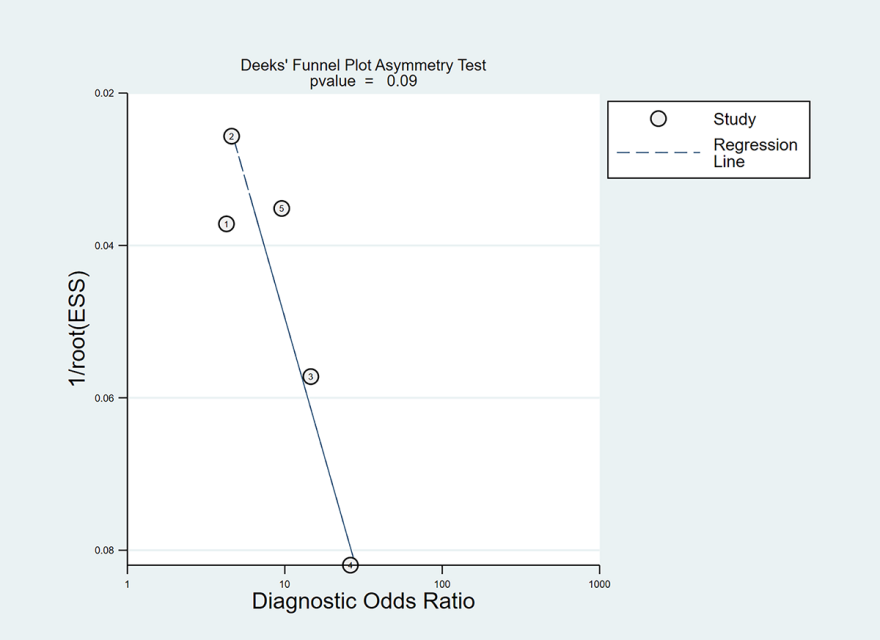


Figure S11 Funnel plot of clinical features


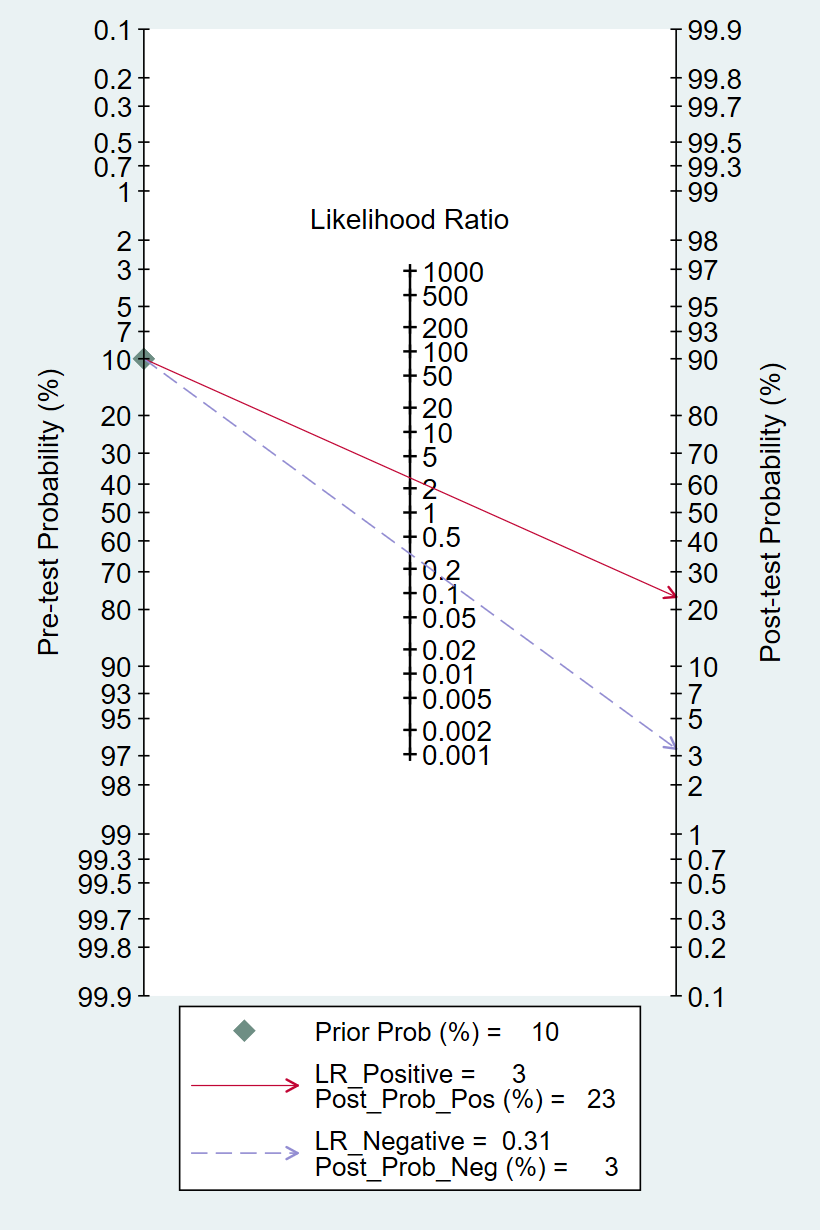


Figure S12 Nomogram of clinical features
